# Notes and News

**Published:** 1893-06-10

**Authors:** 


### Notes and News.

Collected and Communicated.

The Lord Chancellor has consented to preside at the festival of the Homes for Working Boys in London on Thursday evening, July 6th, when a dinner will be given at the Westminster Town Hall, to the 350 boys residing in the eight Homes.

The City of London Truss Society are able to announce a very successful year. The legacies alone during the past twelve months amount to £7,659. The total income for the year was £11,797. This is also the twenty-first year of office of the present able secretary, Mr. John Whittington, and the Society appears to have made a steady but very substantial progress during the whole of that period.

A very sensible little society has been formed in Paris, which might with advantage be imitated in England. This is a medical velocipede club. The establishment of the club is intended to give moral sanction and support to the use of cycles, instead of carriages, by doctors in country districts. The advantage to the struggling practitioner of this economical and sensible method of travelling, and the only barrier—that of etiquette—will soon be removed when cycling is sanctioned from the capital.

Many of our readers are American citizens, or will probably visit the United States during the present year. It may not be uninteresting, therefore, to point out that those who may travel via Liverpool might with advantage use the Midland route from St. Pancras. The Midland Railway run a special express in connection with the Royal Mail steamers, which gets to Liverpool in but little more than four hours. The service is excellent, a luncheon car is attached, much of the scenery is delightful, and the route makes a pleasant variety for the traveller — the English traveller especially. The Midland Railway is universally admitted to have the interests of the public to the fore, and in organising this special service to Liverpool in connection with the Royal Mail steamers from that port to the United States they have taken a step which merits public approval and patronage.

The Metropolitan Drinking Fountains Association held their annual meeting on the 26th ult. at Grosvenor House. Mr. Joseph Fry, in the unexpected absence of the Duke of Westminster, presided. The society is in very urgent need of increased support. The area of the society's work in the metropolitan district covers more than 800 square miles. All the fountains and troughs are examined at least three times per week by men employed by the Association, who travel with this object over 1,500 miles per week. The income for the past year was £4,885, the expenditure £6,363.

Canon Fleming speaks very warmly in favour of the Mothers' Seaside Convalescent Homes at Herne Bay, which we visited unexpectedly, and therefore saw the institution in everyday working order. He found the inmates comfortable and happy, and was much pleased with the arrangements generally. Canon Fleming advocates the establishment of some free beds, and we hope to learn that the Committee have been able to carry out his suggestion before long. Such places of rest and comfort for hard-working mothers are the greatest blessing, and such energy as the Hon. Secretary (Mr. Allen) displays in furthering the interests of the homes must be highly commended.

An important special meeting of the Hospital Sunday Fund was held on Monday last at the Mansion House, the Lord Mayor presiding. Amongst those present were Lord Sandhurst, Sir Sydney H. Waterlow, Dr. Sedgwick Saunders, Canon Ingram, the Rev. Dr. Marks, Mr. H. Fry, Cardinal Vaughan, Mr. A. L. Cohen, and Sir Benjamin Richardson. The Lord Mayor said the work of the hospitals was the most useful and interesting of all the charities they had to deal with in London. The good which was done by the Hospital Sunday Fund was incalculable, and he hoped that the fund would not be allowed to suffer in consequence of the depression now existing. Sir Benjamin Richardson moved the following resolution: "That this meeting pledges itself to use every endeavour to arouse the inhabitants of London to the importance of maintaining the hospitals and medical charities in complete efficiency. In furtherance of this object, it urges the ministers of religion to recommend to their respective congregations the advantage of giving more liberally to hospitals on Hospital Sunday, owing to the mere nominal cost of so collecting, and so as to increase the amount of contributions to £100,000." Lord Sandhurst, in seconding the motion, said he feared that if the public did not support the hospitals in a more substantial way, the day must come when they would have to be subsidised by municipal subvention. He hoped that day would never arrive, but he feared it was inevitable unless larger funds were subscribed. The fund was economically administered, the expenses not exceeding 3½ per cent. of the amount subscribed. In that way the largest possible sums were placed at the disposal of the charities. There was, in his opinion, no nobler object to which they could ask the public to subscribe than the general hospitals of London. Cardinal Vaughan, the Rev. Dr. Marks, and Mr. T. Bryant supported the resolution, which was agreed to. A vote of thanks to the Lord Mayor for presiding concluded the proceedings.

